# Cortical spreading depression and mitochondrial dysfunction with aging: lessons from ethanol abuse

**DOI:** 10.3389/fnagi.2014.00117

**Published:** 2014-06-10

**Authors:** George E. Barreto, Francisco Capani, Ricardo Cabezas

**Affiliations:** ^1^Departamento de Nutrición y Bioquímica, Facultad de Ciencias, Pontificia Universidad JaverianaBogotá D.C., Colombia; ^2^Laboratorio de Citoarquitectura y Plasticidad Neuronal, Facultad de Medicina, Instituto de Investigaciones Cardiológicas Prof. Dr. Alberto C. Taquini (ININCA), UBA-CONICETBuenos Aires, Argentina

**Keywords:** cortical spreading depression, ethanol, mitochondria dysfunction, antioxidants, aging

Chronic consumption of ethanol has a damaging effect in various organs and metabolic functions, including liver, kidney, heart, pancreas, and brain (Sun and Sun, 2001; Bezerra Rde et al., 2005). Additionally, it has been shown that both the acute and chronic ethanol intake induce alteration in voltage-gated channels causing behavioral and electrophysiological changes in the brain (Little, 1999). In the last few years, the effect of ethanol in the electrophysiological phenomenon, known as cortical spreading depression (CSD), has been widely assessed (Sonn and Mayevsky, 2001; Bezerra Rde et al., 2005; Abadie-Guedes et al., 2008) as an important model to predict the damaging effects of ethanol in both young and aged brains.

Cortical spreading depression is a neuronal and glial depolarization wave in gray matter, which is thought to be associated as an upstream event in migraine with aura, spontaneous intracerebral hemorrhage, ischemic injury and brain trauma (Zhou et al., 2010; Dreier, 2011). This phenomenon has a duration of approximately 1 min and very slow propagation (2–5 mm/min) when compared to action potentials, resulting in a suppression of electrical activity and disruption of ion homeostasis (Bennett et al., 2008; Seidel and Shuttleworth, 2011). This depolarization occurs when the extracellular K^+^ concentrations increase above a threshold of 40 mM, causing the CSD wave propagation into adjacent cells. CSD also requires the activation of the N-methyl-D-aspartate (NMDA) subtype of glutamate receptor, which facilitates the spread of the depolarization wave (Eikermann-Haerter and Ayata, 2010).

There are experimental evidences to suggest that various factors influence the brain CSD propagation, including pharmacological substances, age, ethanol abuse and the insufficient ingestion of antioxidants (Abadie-Guedes et al., 2008). To support this idea, previous report has shown that acute ethanol ingestion impairs the CSD propagation when compared to chronic administration of 7, or more days, in which this phenomenon is somehow stimulated (Bezerra Rde et al., 2005). Furthermore, it was previously reported that ethanol-treated rats had alterations in the NADH oxidation cycle and a decrease in energy production and CSD wave frequency (Sonn and Mayevsky, 2001). Acute ethanol administration has been also associated with the inhibition of the Na^+^-K^+^-ATPase pump function, the formation of free oxygen radicals and CSD regulation (Sonn and Mayevsky, 2001). In this aspect, ethanol increases lipid peroxidation in the brain and the serum NO (nitric oxide) levels, probably due to the activation of the inducible nitric oxide synthase (iNOS) by cytokines (Zima et al., 2001). Chronic ethanol administration affects the antioxidant defenses of the brain by reducing the levels of glutathione, peroxidase, α-tocopherol, glutathione reductase and catalase (Bezerra Rde et al., 2005), and the aged brain is particularly affected by this reduced antioxidant scavenging system (Reiter, 1995). Indeed, it is possible that cytoplasmic calcium levels may also be implicated in CSD deflagration (Torrente et al., 2014b). Altogether, these findings suggest that there are multiple mechanisms by which ethanol may damage the brain (Figure 1).

**Figure 1:**
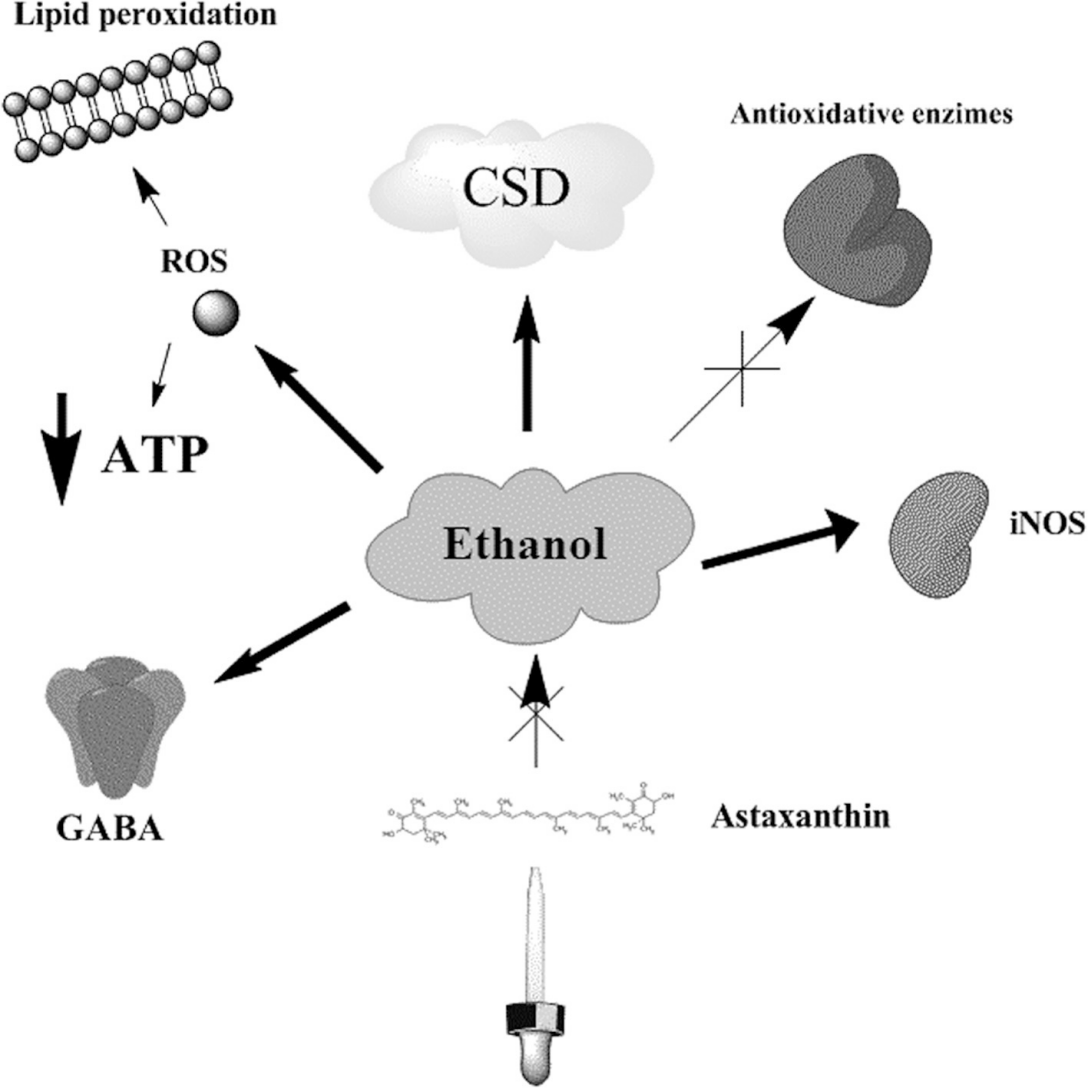
**Ethanol effects on CSD related processes**. Ethanol administration induces the inhibition of Na^+^-K^+^ -ATPase pump, depleting the ATP contents of the cell, and causing the generation of ROS and lipid peroxidation. Both chronic and acute administration of ethanol has impairing effects in CSD velocities, which in turn may reflect important alterations on the bioenergetic metabolism. Additionally, ethanol increases the expression of iNOS and the formation of NO, decreases the levels of antioxidant enzymes, like glutathione reductase and catalase, and promotes the activation of GABA receptor related responses. The administration of astaxanthin counteracts the effects induced by ethanol in CSD velocities.

Although one of the main mechanisms of ethanol-induced brain damage is due to an augmented production of reactive oxygen species (ROS), thus leading to increased CSD propagation, this statement has not been experimentally addressed. Most studies on CSD focused on the electrophysiological effects of ethanol abuse, and their reversion by some protective molecules, such as astaxanthin (Abadie-Guedes et al., 2008, 2012). To address this concern, it is important to point that (i) ethanol facilitates P450 2E1 activity, which metabolizes this compound and generates ROS in the process; and (ii) ethanol increases the levels of free iron in the cell, which also can promote ROS generation. It is possible that most antioxidants may reverse, scavenge, or even impair the production of free radicals and exert protection through this pathway. Ethanol affects the immune system in aged mice, suggesting that this might be one the main mechanisms by which ethanol induces neurodegeneration in aged brains (Drew and Kane, 2013; Kane et al., 2014).

Interestingly, astrocytes have been shown to be more resistant than neurons during ethanol-induced oxidative stress because of their greater content of glutathione (GSH) and other antioxidant enzymes (Watts et al., 2005; Barreto et al., 2011), and these cells may play an important role in CSD (Torrente et al., 2014a). On the other hand, the disruption of astrocyte oxidative metabolism, which results in ATP levels decline, has been shown to increase the rate of spreading depression in rat cortex (Largo et al., 1997; Seidel and Shuttleworth, 2011), suggesting that metabolic alterations of astrocytes are an important regulator of CSD events, especially during normal aging process (Farkas et al., 2011; Paradies et al., 2013; Yin et al., 2014). ATP depletion is likely to occur due to the oxidation of ethanol to acetaldehyde catalyzed by alcohol/dehydrogenase containing the coenzyme NAD^+^. The acetaldehyde is further oxidized to acetic acid and finally CO_2_ and water through the citric acid cycle. A number of metabolic effects from ethanol are directly linked to the production of an excess of both NADH and acetaldehyde. In this context, more NADH will dislocate the equilibrium of converting glucose to piruvate for energy production toward a overproduction of lactate and malate from piruvate. The increased accumulation of acetaldehyde by reduced conversion of this product into acetic acid—both processes impair mitochondrial functions and energetic metabolism—results in decreased ATP production. It is important to point that brain metabolism in the aged brain is somehow impaired, therefore the damaging effects of CSD are augmented (Farkas et al., 2011; Batista-De-Oliveira et al., 2012).

Following a cortical spreading depression event, metabolic recovery is compromised, as glucose and glycogen levels are significantly decreased and lactate levels are found increased (anaerobic shuttle), thus leading to intense depolarization and cell death (Selman et al., 2004). In this context, we hypothesize that aging influences brain electrophysiological functioning due to reduced metabolic shuttle and antioxidant defense, and the impairment of both energy metabolism and redox homeostasis are a hallmark of brain aging (Yin et al., 2014). These observations are particularly seen in older people with alcoholism problems. A previous study reported that brain tissue loss was found increased in older subjects with alcoholism compared to younger subjects with alcoholism (Oscar-Berman et al., 1997), and this may accelerate normal aging process or cause premature aging of the brain. Importantly, ethanol was shown to impair vasodilatation enzymes such as nNOS (neuronal nitric oxide synthase) and eNOS (endothelial nitric oxide synthase) by the augmentation of ROS, and this was associated with an increase in ischemic brain damage in elder individuals (Sun et al., 2006).

To counteract this damaging effect of ethanol on aged brain, and following CSD, different antioxidant molecules and enzymes, such as α-tocopherol, ascorbic acid, peroxiredoxin, and astaxanthin are shown to be neuroprotective against ethanol intake in animal models (Bezerra Rde et al., 2005; Lee et al., 2010; Zhu et al., 2012). The carotenoid astaxanthin (3,3′-dihydroxy-β,β-carotene-4,4′-dione) has important metabolic functions, including its conversion to vitamin A, enhancement of immune response, inhibition of NO and lipopolysaccaride (LPS)-induced inflammation, scavenging action on reactive oxygen species, and protection of gastric mucosa against ethanol-induced injuries (Kim et al., 2005; Lee et al., 2010). Astaxanthin was also shown to protect against induced cerebral ischemia in rats by the inhibition on iNOS and induce the expression of Hsp32 and Hsp70, demonstrating a decrease in oxidative stress without affecting cell viability. This information supports the idea of an oxidative mechanism by which astaxanthin may be exerting a protective effect on ethanol induced damage. On this perspective, new findings on the antioxidant effects of astaxanthin and their relationship with CSD were previously reported (Abadie-Guedes et al., 2012). In this study, the antagonistic effects of acute ethanol consumption on CSD propagation in two groups of rats with different ages were assessed. Their main results showed that CSD outcome was counteracted by astaxanthin administration. Age of animals and time of ethanol/astaxantin administration are the key features, and age related effects of ethanol in the brain should be further addressed in additional experiments related with ROS production in both brain and blood of the treated animals, as well as the expression of antioxidant enzymes such as iNOs or SOD (superoxide dismutase). In this point of view, some hypotheses must be raised: (i) Perhaps, the antioxidant properties of astaxanthin on the scavenging of ROS could explain the reversion of ethanol effect on CSD velocities; and (ii) alternatively, it is possible that ethanol action on CSD involves an interaction with the GABA-chloride ion channels and the NMDA receptors, causing a suppression of nerve cell activity, through ionic modulation (Sonn and Mayevsky, 2001; Criswell et al., 2003). Whether or not this relationship between astaxanthin and the GABA is clear, this is a concern that deserves further attention. Moreover, it is important to address the possible neuroprotective effects of other antioxidants on the modulation of CSD in the setting of a pathophysiological condition, as is migraine with aura, stroke or brain injury, and also in the normal aging process.

## Conflict of interest statement

The authors declare that the research was conducted in the absence of any commercial or financial relationships that could be construed as a potential conflict of interest.
